# Seroprevalence against Diphtheria in Pregnant Women and Newborns in Colombia: New Arguments to Promote Maternal Immunization

**DOI:** 10.3390/vaccines10030458

**Published:** 2022-03-17

**Authors:** Laura María Rivera-Santamaría, Doracelly Hincapié-Palacio, Jesús Ochoa, Felipe Vargas-Restrepo, Marta C. Ospina, Seti Buitrago-Giraldo

**Affiliations:** 1 “Hector Abad Gómez” National Faculty of Public Health, Universidad de Antioquia UdeA, Medellin 050010, Colombia; doracelly.hincapie@udea.edu.co (D.H.-P.); jesus.ochoa@udea.edu.co (J.O.); felipe.vargasr@udea.edu.co (F.V.-R.); 2 Laboratory of Public Health of the Regional Secretariat of Health and Social Protection of Antioquia, Medellin 050010, Colombia; martha.ospina@antioquia.gov.co (M.C.O.); seti.buitrago@antioquia.gov.co (S.B.-G.)

**Keywords:** seroepidemiological studies, diphtheria-tetanus-acellular pertussis vaccines, immunization, Colombia

## Abstract

The tetanus toxoid, reduced diphtheria toxoid, and acellular pertussis (Tdap) vaccine is recommended during pregnancy for neonatal protection against pertussis, although little is known of the protection it provides against diphtheria. The work used a cross-sectional design to estimate seroprevalence against diphtheria in 805 pregnant women with ≥37 gestation weeks and their newborns whose deliveries were attended in eight hospitals randomly chosen from a subregion of Antioquia, Colombia and to explore factors related with maternal protection. Levels of IgG antibodies were determined by using a commercial enzyme-linked immunosorbent assay test. Placental transfer of antibodies and crude and adjusted prevalence ratio (aPR) were analyzed to describe factors related with maternal protection against diphtheria. Protection against diphtheria was observed in 91.7% (95% CI 90.3–93.0) of the pregnant women and 93.1% (95% CI 91.7–94.4) of newborns, whose antibody levels were positively correlated (Spearman’s r = 0.769; *p* = 0.000). Maternal protection could be influenced by having been vaccinated during the current pregnancy (aPR 0.85, 95% CI: 0.82–0.93). The protective effect of vaccination during pregnancy and the efficiency of maternal antibody transfers were detected. Public health efforts should focus on increasing Tdap vaccination during each pregnancy to protect mothers and newborns against diphtheria.

## 1. Introduction

Diphtheria is a contagious disease caused by exotoxin-producing bacteria of the *Corynebacterium diphtheriae* species, which can damage the upper respiratory tract, myocardium, and other tissues. As complications, myocarditis and neuritis are more frequently reported [1].

Diphtheria is considered a threat to public health globally with special concern in lower income regions, with political and social instability. Diphtheria was one of the main causes of global morbidity and mortality in the infant population before the introduction of the vaccine. Subsequently, cases have been described (sporadically and in outbreaks) in countries that have experienced crises in their health systems and vaccination programs [2]. A diphtheria epidemic was described in the 1990s, originating in Russia and later spreading to the newly independent states of the Soviet Union [3]. Since 2000, the disease has varied in the number of cases reported. The average number of cases reported globally per year during the 2013–2017 period was 6582—representing a 37% increase compared to the average from 2008 to 2012 [4].

In the Americas, according to the Pan American Health Organization, between 2018 and 2019, confirmed cases of diphtheria were reported in Colombia, Haiti, and Venezuela. In Haiti, between 2018 and 2020, there have been 165 confirmed cases and an annual fatality rate between 14% and 22%. In Venezuela, a diphtheria outbreak began in 2016. During this period, 1785 confirmed cases and 292 deaths have been reported, with the highest fatality rate in the 5-year age group. Additionally, there was a high incidence of diphtheria cases in countries, such as Brazil and the Dominican Republic [5].

To our knowledge, few studies conducted in the Americas have investigated the impact of maternal vaccination on immunity against diphtheria. A previous study in Colombia [6,7] corroborated the transfer of maternal antibodies against pertussis to newborns, depending on the vaccination status of pregnant women with maternal tetanus toxoid, reduced diphtheria toxoid, acellular pertussis (Tdap) vaccine in Medellín, the second city that started maternal vaccination early in 2013.

The analysis of the protective effect of Tdap vaccination during each pregnancy, based on scientific evidence, has been recognized as the best means to reduce hesitance about vaccination and ensure vaccination during the course of each pregnancy [8]. Colombia, like other American countries and the rest of the world, needs to increase coverage and acceptance of vaccinating pregnant women with Tdap, which has decreased during the last year after the COVID-19 pandemic [9]. This can be achieved if local socioeconomic conditions of mothers, families, and their communities are also taken into account [10].

In Colombia, vaccination against diphtheria, tetanus, and pertussis (DTwP) began in the 1970s. The routine vaccination schedule includes three initial doses of DTwP in the pentavalent vaccine (DTwP, hepatitis B, and Hemophilus influenza type b) for children between 6 and 23 months, with an interval of eight weeks between doses, and two boosters in trivalent DTwP presentation for children from 1 to 6 years of age [11,12]. Vaccination programs targeted women of childbearing age with five doses of Td vaccine and a booster every 10 years. Starting in 2013, the vaccination of pregnant women with one dose of Tdap during each pregnancy began, given the pertussis epidemic from 2010 to 2013 [12,13].

The objective of this work was to determine the seroprevalence of IgG antibodies against diphtheria in pregnant women, to verify antibody transfer from a pregnant woman to her newborn through the umbilical cord, and further to evaluate factors related with maternal protection against diphtheria.

## 2. Materials and Methods

This cross-sectional study assessed the anti-diphtheria immunity in pregnant women and umbilical cord. Additionally, we analyzed placental transfer of antibodies and factors related with maternal protection.

### 2.1. Participants

The samples processed came from the follow up of a cohort of pregnant women vaccinated and unvaccinated with Tdap conducted among pregnant women and their newborns, as part of the departmental sero-surveillance program of Antioquia. The study population comprised pregnant women living in Medellín and metropolitan area, Colombia, whose delivery was attended in eight hospitals randomly chosen. The sample was stratified by municipality according to Tdap vaccination coverage, health insurance affiliation, and deliveries attended in hospitals, as described [6].

In summary, pregnant women with ≥37 weeks and vaginal delivery were included. Those with multiple gestation, those who had a fever within 72 h prior to recruitment, those who had serious decompensated illnesses that required attention in the intensive care unit, or those who had advanced labor were excluded.

### 2.2. Procedures

The pregnant women were recruited at the time of delivery between December 2015 and April 2016 after random selection of a representative sample of hospitals that attended deliveries and Medellín and its metropolitan area. A blood sample was taken from the pregnant woman and the umbilical cord. The samples were preserved at −80 °C in the serum bank of the departmental sero-surveillance program at the Departmental Laboratory of Public Health, Sectional Secretariat of Health of Antioquia, and processed after training and standardization for purposes of this study.

Ethics approval was obtained from the Ethics Committee of the National Faculty of Public Health (act code: 021, February 2019) and the participating hospitals. National and international ethical guidelines were followed. Written informed consent and assent was obtained with authorization from the guardian or legal representative of pregnant women under 18 years of age, including voluntary participation in a face-to-face interview and multipurpose blood sample collection.

### 2.3. Variables

A survey was completed for each pregnant woman regarding socio-demographic variables, including age, date of birth, urban or rural area, socioeconomic level (low being 1 to 3 and medium-high being 4 to 6), health insurance coverage, marital status, years of schooling, number of people in the household, rooms in the home, and overcrowding (more than three people per room). Previous immunization against diphtheria during childhood along with current and previous pregnancy was registered based on the vaccination card and this was corroborated by using a centralized database managed by the local health authority (“PaiWeb”).

### 2.4. Diphtheria Assay

IgG antibodies specific for *Corynebacterium diphtheriae* toxin were determined by using the ELISA test Corynebacterium diphtheriae toxin IgG NovaTec (NovaLisa)™ following the manufacturer’s instructions (Product number: CORG0090, NovaTec Immunodiagnostica GmbH Technologie & Waldpark, Germany) [14].

To interpret the results, the classification ranges suggested by the manufacturer were used. Antibody levels were quantified in International Units per milliliter (IU/mL). The results were classified as <0.01 IU/mL as unprotected, between 0.01–0.09 IU/mL uncertain protection and ≥0.1 IU/mL as protected.

### 2.5. Statistical Methods

The weighted prevalence of IgG antibodies to diphtheria and 95% confidence intervals were estimated by following a complex sampling, *p* ≤ 0.05 was considered statistically significant. To test for differences in sociodemographic variables between the vaccinated and unvaccinated groups, χ^2^ and Fisher’s exact tests were performed. Correlation between maternal and cord blood anti-diphtheria antibodies was determined by using Spearman’s correlation test. Bivariate and binomial regression analysis were performed to evaluate the influence of variables on maternal protection against diphtheria, categorized as protected (≥0.1 IU/mL) and unprotected (<0.09 IU/mL).

## 3. Results

### 3.1. Patients

In our study, 805 pregnant women participated. Blood samples were analyzed from 803 (99.75%) pregnant women and 755 (93.79%) umbilical cord samples.

The pregnant women had a median age of 23 years (IQR:13–43). The median gestational age at delivery was 39 weeks (IQR: 37–41). Most lived in urban areas, were married or in a common-law union, belonged to the low socioeconomic stratum, had households with fewer than three people per room, and had studied for at least 11 years (two had no education whatsoever and 7.5% had only studied until some grades in primary school). In terms of health insurance, 761 (94.5%) were affiliated, while 44 (5.5%) were not (Table 1).

### 3.2. Tdap Vaccination Status during the Pregnancy

This study included 539 (67%) Tdap vaccinated pregnant women during the current pregnancy and 266 (37%) not vaccinated. We observed that 9 (4.5%) had not been vaccinated during pregnancy or infancy.

Regarding the history of vaccination during pregnancy, 75 (63%) were not vaccinated during any of the pregnancies and the vaccination was not repeated in each pregnancy in 44 (37%) of the pregnant women.

The unvaccinated pregnant women were mainly between 13 and 23 years of age, did not live in overcrowded housing conditions, were not affiliated to the contributive health insurance, and had up to 11 years of schooling.

Significant differences were observed in antibody levels by age (median value 13–23 age: 389.27; 24–43 age: 412.71; Mann–Whitney U test: 75 506 *p* value: 0.013) and Tdap vaccination during the current pregnancy (median value vaccinated: 428.61; not vaccinated: 355.14; Mann–Whitney U test: 61 447.5; *p*-value: 0.000).

### 3.3. Weighted Prevalence of IgG Antibodies to Diphtheria

Regarding the weighted prevalence of IgG antibodies to diphtheria, protection was found in 91.4% (95% CI 90.3–93.0) of pregnant women and 93.1% (95% CI 91.7–94.4) of umbilical cord samples. No protection against diphtheria was found in 8.3% (95% CI 7.0–9.7) of pregnant women and 6.9% (95% CI 5.7–8.3) of umbilical cord samples.

In pregnant women, protection was found in 95.9% (*n* = 517) of those vaccinated (geometric mean = 0.140; IQR: 0.148) and 82.3% (*n* = 219) of those not vaccinated (geometric mean = 0.10; IQR: 0.149). Similarly, in the umbilical cord, protection was found in 91.3% (*n* = 492) of those vaccinated (geometric mean = 0.0; IQR: 0.15) and 78.6% (*n* = 209) of those not vaccinated (geometric mean = 0.115; IQR: 0.149).

A positive correlation was observed between diphtheria IgG levels of pregnant women and umbilical cord (Spearman’s r = 0.769; *p* < 0.05).

Table 2 shows the concordance of the prevalence of protection of the pregnant woman and the cord. Protection of both coincided in 84.4% (95% CI 80.5–87.6) and non-protection coincided in 1.4 (95% CI 0.9–2.1).

**Figure 1:**
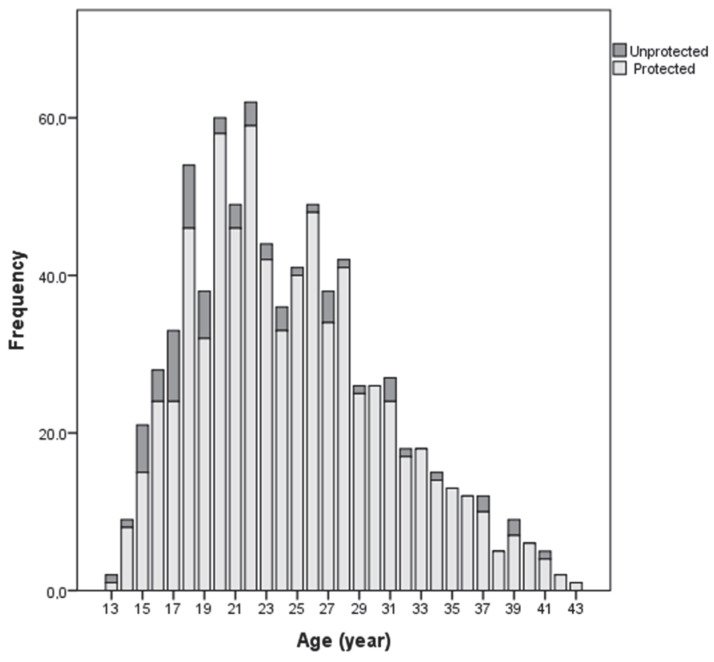
IgG antibodies against diphtheria in pregnant women, according to age and protection status.

Figure 1 shows a greater distribution of unprotected pregnant women in younger pregnant women (geometric mean = 0.058; IQR: 0.063). No differences were observed in levels of antibodies, according to the gestational week at vaccination in the protected pregnant women, both in their samples and in the umbilical cord. In the unprotected pregnant women, the geometric mean of antibodies was higher if they were vaccinated at 38–40 weeks (geometric mean = 0.058; IQR: 0.063, pregnant sample) or at 32–34 weeks (geometric mean = 0.084; IQR: 0.014, cord blood sample).

**Figure 2:**
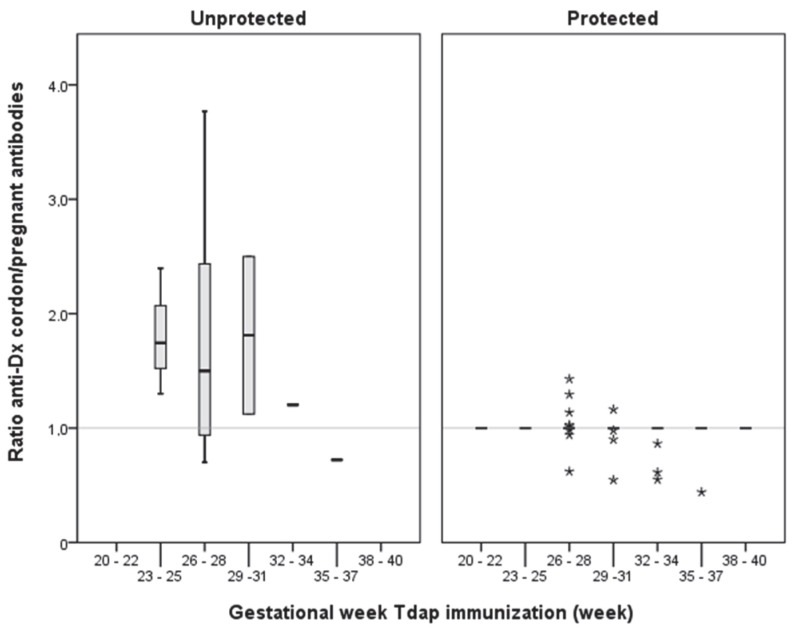
Ratio of cord/pregnant woman antibodies, according to the gestational week to immunization by using the Tdap vaccine and maternal protection status. Asterisk symbols are outliers, that is, values more extreme than the expected variation. The horizontal line shows the ratio of cord antibodies in relation to maternal antibodies, equal to unity, considered a reference value for efficient placental transfer.

Antibody transfer was efficient in all cases, except in four unprotected pregnant women in which the ratio of cord to pregnant women antibodies was <1, although this analysis may be limited due to the low number of pregnant women not protected (*n* = 19) (Figure 2).

### 3.4. Factors That May Influence the Protection of Pregnant Women

Some factors may influence the protection of pregnant women. As shown in Table 3, the prevalence of protection was significantly higher in pregnant women vaccinated with Tdap during the current pregnancy.

## 4. Discussion

In Colombia, vaccination against diphtheria, tetanus, and pertussis (DTwP) has reduced notably the incidence and mortality of these diseases [15]. In the country, from 2000 to 2019, 26 cases of diphtheria were confirmed, with a report of three deaths due to diphtheria in 2018 [16].

Our study found protection against diphtheria in 91.7% (95% CI: 90.3–93.0) of pregnant women and in 93.1% (95% CI: 91.7–94.3) of newborns and significant correlation of maternal and umbilical cord antibodies after childhood vaccination with DTwP began in the mid-1970s and maternal vaccination with Tdap began in 2013.

These findings coincide with similar studies. In the Netherlands between 2002 and 2006, researchers recruited 197 pregnant women and their newborns in a hospital from the region. They analyzed the seroprevalence of diphtheria, among other diseases, by an ELISA test. Protection was present in 99% of pregnant women and 96% of newborns; antibody transfer was 160%. In that region, the regular vaccination program began in 1993, including three doses of DTwP, plus one booster dose. The diphtheria vaccination during pregnancy was not included in the routine vaccination schedule when the study was conducted [17].

A study from the United States recruited 58 pairs of mothers and cord samples between 2011 and 2017. The pregnant women received Tdap vaccine before or during pregnancy. It was found that 100% of the babies born to mothers who received Tdap during pregnancy were protected against diphtheria, while 62.5% of children from mothers who were vaccinate before pregnancy had different levels of protective antibodies. The regular childhood immunization schedules have 3 plus 2 booster doses of DTaP since 1997 and Tdap maternal vaccination has been included in their regular vaccination schedule [18].

In 2010–2011, researchers in Nigeria conducted a study at a university hospital, including 231 pregnant completely healthy women at term who had selected vaginal delivery. They determined IgG antibody level for diphtheria by using an ELISA test (IBL International). The Nigerian vaccination program began in 1974, including three doses of the DTwP vaccine, without boosters or maternal vaccinations with Tdap, at the time of the study; 133 (57.6%) pregnant women and 123 (53.2%) umbilical cord samples had safe protection. As in our study, they detected a strong positive correlation between maternal and umbilical cord antibodies, but detected a high proportion of mothers (*n* = 80, 39%) and neonates (*n* = 107, 46.3%) without protection against diphtheria [19].

In the region of the Americas, other studies to determine protection against diphtheria have been conducted in the overall population rather than in pregnant women. In Bonaire, in 78.3% (95% CI: 75.2–81.3) of the population aged 0 to 90 years had protection, while in Brazil, 84% of individuals aged 60 or over had complete protection against diphtheria [20,21].

Our study corroborated the effect of Tdap vaccination on maternal protection against diphtheria; 95.9% (*n* = 517) of the vaccinated pregnant women during the current pregnancy and 82.3% (*n* = 219) of the unvaccinated pregnant women had protective antibodies.

Protection against diphtheria in unvaccinated pregnant women during pregnancy may indicate the magnitude of previous natural exposure, taking into account that about 5% (*n* = 13) of unvaccinated pregnant women were born before the start of vaccination with DTwP, when the incidence of diphtheria was higher. The protection observed may also be due to previous vaccination during childhood or previous pregnancies, although vaccination history was only obtained in 74.7% and 39.5% of pregnant women, respectively. There are no known publications on the magnitude of vaccine rejection and hesitation in Colombia, although the presence of different types of barriers during childhood vaccination has been reported, including barriers related to conditions of parents or caregivers (24.4%), vaccination services (19.7%), and health centers (18%) [22].

The trans-placental efficiency of antibodies according to the time of vaccination during gestation has been well-documented in the case of pertussis, but less in diphtheria [18]. In our study, protection against diphtheria was achieved independent of the gestational week of vaccination and the transfer of antibodies was efficient, even in unprotected pregnant women.

In our study, Tdap vaccination during pregnancy was a factor that could influence the maternal protection against diphtheria. Of the pregnant women vaccinated with Tdap during the current gestation, less than 1% (*n* = 3) did not have protective antibodies and 3.2% (*n* = 17) had antibodies—but protection was uncertain. The effectiveness of maternal pertussis vaccination has been thoroughly studied [23], but there is a lack of studies aimed at evaluating the effectiveness of diphtheria through the follow-up of cohorts of children, which was not possible in this study [24].

Other studies have identified differences in protection against diphtheria according to age and gender [25]. In our study, the prevalence of protection against diphtheria was higher in people aged 24 to 43, compared with younger people aged 13 to 23, although not statistically significant. This may have been influenced by the higher probability of natural exposure with age and the higher frequency of vaccination during pregnancy in this group. In younger pregnant women, educational approaches on the importance of vaccination should agree based on their perspectives and ways of life. It would also be convenient to monitor the duration of immunity following long-term cohorts, given the possibility of the loss of vaccine immunity over time, which could suggest the presence of unprotected pregnant women in this younger group (Figure 1).

### Strengths and Limitations

The pregnant women studied were predominantly from the lower-middle stratum, similar to the reference population, but our study could have limitations to detect significant differences for these variables because the study was not designed to detect differences by social status. In Colombia, no previous diphtheria seroprevalence studies are known, but the effect of socioeconomic characteristics on childhood vaccination has been analyzed. A study analyzed municipal vaccination coverage, the timing of vaccination, and factors that could influence these indicators. The authors conducted a population survey of children under six years of age, whose homes were randomly chosen from a two-stage sample of 80 municipalities in the country. They analyzed disparities in immunization, identifying that the mother’s schooling, older age, having more siblings, low socioeconomic status, and not having health insurance were associated with low immunization [26].

This study had limitations in determining whether the protection conferred was long-term due to the maximum detection limit of 0.150 of the test used. This does not affect the seroprevalence estimated, but rather prevents individual guidance on the need for booster vaccines in pregnant women without protection or uncertainty in the protection duration. The ELISA test used in our study has been previously analyzed and used in population studies to measure the levels of antibodies to diphtheria [27]. Studies found good consistency (>80%) in the results when compared with an international standard for diphtheria antitoxin. When compared with other tests for the detection of IgG against *Corynebacterium diphtheriae* toxin, its relative sensitivity and specificity were high, and it had the ability to detect lower results (below the WHO recommended level of protection 0.1 IU/mL) compared with other tests [14,28].

## 5. Conclusions

This study was able to detect a prevalence of protection of pregnant women and umbilical cords > 90%, efficient placental transfer of antibodies, and greater protection if the pregnant women had been vaccinated during the current pregnancy.

In Latin America, there is concern about the resurgence of diphtheria due to the difficulty of obtaining optimal vaccination coverage, which has been aggravated by the reduced or delayed access to vaccination services during the COVID-19 pandemic [29] and the recent presence of outbreaks and cases in unvaccinated children. To circumvent the spread of diphtheria, measures are needed to promote and facilitate immunization with Tdap of pregnant women during each pregnancy and ensure full immunization of children.

## Figures and Tables

**Table 1:** Sociodemographic variables of pregnant women.

Variables	*n* (%)
Age (years)	
13–23	404 (50.2)
24–43	401 (49.8)
Marital status	
Married/common law	586 (71.9)
Single/divorced	219 (28.1)
Area	
Rural	40 (5.0)
Urban	765 (95)
Socioeconomic level	
1 to 3 (low)	708 (95.3)
4 to 6 (high)	35 (4.7)
Three or more people per room	
Yes	26 (3.2)
No	779 (96.8)
Health insurance	
Contributive	385 (47.8)
Other affiliation	420 (52.2)
Years of schooling	
1–11	614 (76.7)
12–more	187 (23.3)
Birth before 1980 (start of DPT vaccination)	
Yes	49 (6.1)
No	756 (93.9)
Childhood immunization	
Yes	582 (96.8)
No	19 (3.2)
Vaccination during previous pregnancy	
Yes	83 (26.1)
No	235 (73.9)
Protective antibodies (pregnant)	
Yes	736 (91.4)
No	12 (1.5)
Yes, but without certainty of protection	54 (6.7)
Missing data	3 (0.4)
Protective antibodies (umbilical cord)	
Yes	701
No	12
Yes, but without certainty of protection	42
Missing data	50

*n* = 805.

**Table 2:** Concordance in proportion of IgG antibodies against diphtheria in pregnant women and umbilical cord.

Pregnant Proportion (95% CI) *n*	Umbilical Cord Proportion (95%CI) *n*			
	Protected	Unprotected	Protection Uncertain	Total
Protected	84.4 (80.5–87.6) 685	0.1 (0–0.3) 1	1.3 (0.9–1.9) 9	736 (91.0) 695
Unprotected	-	1.4 (0.9–2.1) 10	-	1.8 (1.2–1.8) 10
Protection uncertain	1.9 (1.2–3.1) 15	0.1 (0–0.4) 1	4.3 (3.2–5.8) 33	7.0 (5.9–82) 49
Total	86.5 (83.2–89.2) 701	1.6 (1.0–2.5) 12	5.6 (4.4–7.2) 42	(100) 805

Missing data: pregnant women = 3; umbilical cord = 50.

**Table 3:** Characteristics of pregnant women that could influence their protection against diphtheria.

Variable	Protected *n*	Not protected *n*	Crude PR (95% CI)	Adjusted PR (95% CI)
Age (years)				
13–23	375	50	1.06 (1.02–1.11)	1.01 (0.97–1.06)
24–43	395	26	1	1
Vaccination during the current pregnancy				
Yes	532	22	1	1
No	238	54	0.85 (0.81–0.90)	0.87 (0.82–0.93)
Health Insurance				
Contributive	368	30	1.02 (0.97–1.06)	1.00 (0.95–1.05)
Other affiliation	402	46	1	1
Years of schooling				
1–11	557	66	0.96 (0.92–1.00)	0.98 (0.94–1.03)
12–more	144	10	1	1
Socioeconomic level				
1 to 3 (low)	678	34	0.94 (0.89–1.00)	0.99 (0.92–1.06)
4 to 6 (high)	63	1		-

## Data Availability

The data presented in this study are available on request from the corresponding author. The data are not publicly available due to ethical and privacy issues.
